# Caffeine and Gastric Emptying Time in Very Preterm Neonates

**DOI:** 10.3390/jcm9061676

**Published:** 2020-06-01

**Authors:** Antonios K. Gounaris, Ioanna N. Grivea, Maria Baltogianni, Eleni Gounari, George Antonogeorgos, Fedra Kokori, Polytimi Panagiotounakou, Martha Theodoraki, Aikaterini Konstantinidi, Rozeta Sokou

**Affiliations:** 1 Neonatal Clinic-NICU, University General Hospital, 413 34 Larissa, Greece; ioanna.grivea@gmail.com; 2 Neonatal Clinic-NICU, University General Hospital, 451 10 Ioannina, Greece; mbalt@doctors.org.uk; 3 NICU, Royal Alexandra Children’s Hospital Brighton, Eastern Road, Brighton, East Sussex BN2 5BE, UK; elenigounari@gmail.com; 4 Neonatal Clinic-NICU, General Hospital “Agios Panteleimon”, 18454 Piraeus, Greece; gantonogeorgos@gmail.com (G.A.); ppppolytimi04@gmail.com (P.P.); anastasiosmmr@yahoo.gr (M.T.); kmaronia@gmail.com (A.K.); sokourozeta@yahoo.gr (R.S.); 5 Department of Radiology, General Hospital “Agios Panteleimon”, 18454 Piraeus, Greece; gkandilis@yahoo.gr

**Keywords:** caffeine, methylxanthines, gastric motility, ACSA, gastric emptying time, very preterm neonates

## Abstract

Background: Caffeine has been commonly used for prevention and treatment of apnea-related symptoms in premature infants. However, its side effects have not been thoroughly studied. We investigated whether caffeine affects gastric motility in very-preterm (VP) neonates. Methods: The study is a randomized crossover clinical trial. Twenty-two neonates with mean birth weight (BW) (standard deviation—SD) 1077 (229) g and mean gestational age (GA) (SD) 28.6 (2.1) weeks were recruited. Each neonate had its gastric emptying time checked twice with ultrasound assessment of changes in antral cross sectional area (ACSA). All neonates were sequentially allocated to the caffeine group (A) and the control group (B). Complications from the gastrointestinal tract were documented throughout the study. Results: Statistically significant difference was found with regards to the gastric emptying time [median, (range)] between caffeine and control group (*p* = 0.040). Additionally, in the neonates with BW 1000–1500 g and GA ≥ 28 weeks, the gastric emptying time (minutes) was significantly longer during caffeine treatment [44.5 (36–68.2)] and [40 (34.5–66.5)] respectively, as compared to the gastric emptying time during no caffeine treatment [27 (24.2–30)] (*p* = 0.002) and [27 (24.5–30)] (*p* = 0.001). The incidence of gastrointestinal (GI) complications was significantly greater in neonates receiving caffeine [6 (27.%)] as compared with those without caffeine treatment [1 (4.6%)] (*p* = 0.039). Conclusions: During caffeine treatment, a significantly delayed gastric emptying time was noted in all study neonates, especially in these with BW 1000–1500 g and those with GA ≥ 28 weeks. Further larger studies are necessary in order to confirm this interesting finding.

## 1. Introduction

Methylxanthines such as aminophylline, theophylline and caffeine have been used over the last decades as respiratory stimulants for the treatment of apnea of prematurity (AOP) [1]. Caffeine is the most commonly administered methylxanthine in NICUs since 2006 [2] and it has been used for the prevention and treatment of apnea-related symptoms in preterm infants with gestational age (GA) less than 34 weeks, especially in the first 10 days of life [3,4]. A large prospective multicenter study of very premature infants, showed that caffeine had beneficial effects not only on the AOP, but also on the early and late morbidity of these infants. The results of this study revealed that very preterm neonates who received caffeine treatment for AOP had a lower incidence of bronchopulmonary dysplasia (BPD), improved survival and a better neurodevelopmental outcome at the age of 18–24 months [5,6]. However, in the same cohort it was demonstrated that the neonates who received caffeine therapy had no significantly improved disability-free survival rate at 5 years of age [6].

A recent retrospective study concluded that premature neonates with birth weight (BW) up to 1250 g who received early (before the third day of life) caffeine therapy had improved neonatal outcomes including survival, BPD and patent ductus arteriosus [4]. The beneficial effects of caffeine use reported in the current literature, have encouraged neonatologists to use it more frequently when treating very preterm (VP) infants.

However, methylxanthines and especially caffeine have been associated with adverse effects. As shown in a recent study on rodents, the parallel administration of caffeine and sedative/anesthetic may augment neurotoxic action by triggering widespread apoptosis in the developing brains [7]. In another recent study, Fazeli W et al. [8] showed detrimental effects of caffeine exposure to rodent brain development. Caffeine administration to newborn rats at a dose comparable to the one therapeutically used for preterm neonates impairs lower esophageal sphincter (LES) and gastrointestinal motor function [9].

In neonates Schmidt et al. [2] reported poor weight gain during the first three weeks after initiation of caffeine treatment in the studied neonates when compared to the control group. This finding was attributed to the increased metabolic rate and increased oxygen consumption caused by the drug.

Gounaris et al. [10] reported that gastric emptying in very low birth weight (VLBW) infants that received theophylline during their first three weeks of life was delayed compared to the control group. In a recent meta-analysis 10% of the VLBW neonates treated with caffeine developed feeding intolerance [11]. Belen-Rivas et al. [12] found that systemic antimicrobials and caffeine were the drugs most commonly associated with adverse reactions in very preterm neonates.

Despite the large number of studies on the benefits of caffeine, data around its effects on the gastrointestinal tract of VP infants and particularly on gastric emptying time are scarce. We hypothesized that caffeine, may affect gastric motility and gastric emptying time in VP infants.

Primary objective of our study was to investigate the effect of caffeine on the gastric emptying time of VP neonates. Secondary objectives were (a) to assess if the effect of caffeine on gastric emptying time differs in our premature infants depending on their BW and GA, (b) to examine the occurrence of gastrointestinal (GI) complications during caffeine treatment.

## 2. Material and Methods

### 2.1. Study Design-Setting

This single-center, randomized, crossover clinical trial was conducted during over a 6 months period at a tertiary-level 26 bed Neonatal Intensive Care Unit (NICU) in Nikaia General Hospital “Aghios Panteleimon”, Piraeus, Greece.

### 2.2. Participants

**Figure 1:**
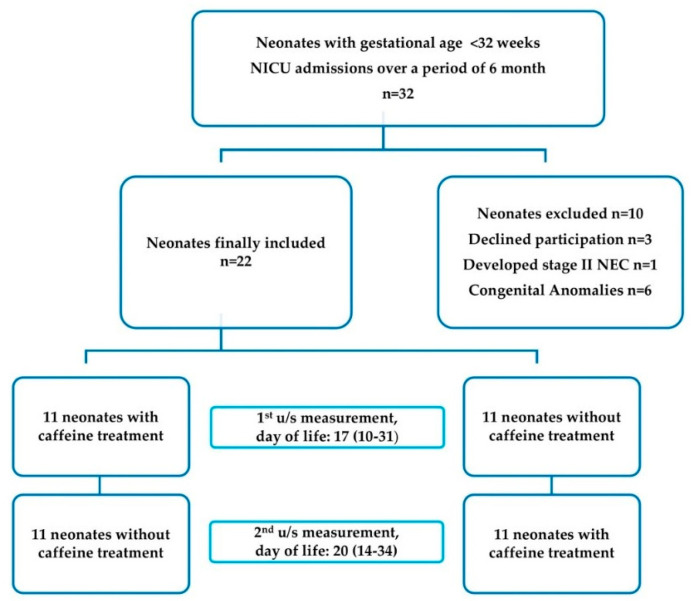
Flow chart of study population.

The frequency of apnea of prematurity correlates inversely to gestational age and the more vulnerable population for its occurrence is that of preterm neonates with gestational age < 32 weeks [13,14]. Hence, we identified 32 preterm neonates appropriate for gestational age with BW less than 1500 g and GA < 32 weeks requiring caffeine treatment. Neonates with congenital anomalies, intrauterine growth retardation (IUGR) or necrotizing enterocolitis (NEC) stage II or III were excluded from the study. The final sample consisted of 22 neonates with a mean BW (SD) of 1077 (229) g and a mean GA (SD) of 28.6 (2.1) weeks. Data regarding the recruitment process of study population are presented in flow chart (Figure 1).

All procedure performed in this study were in accordance with the ethical research committee and with the 1964 Helsinki declaration. Following a detailed discussion regarding the study’s aims and process, parents signed a written consent form. The “Aghios Panteleimon” Hospital Ethics Committee approved the study (15.07.2015, 32/4).

### 2.3. Study Protocol

A crossover study design was implemented in order to reduce the impact of the postmenstrual age on the maturation of gastrointestinal tract, as this could be a confounding factor for the assessment of gastric emptying time. Because gastric emptying would be assessed in each neonate twice during the study period we aimed for the measurements to be only few days apart in order to ensure that each neonate was on a similar postmenstrual age during each of the two measurements.

The randomization was performed with sealed envelopes during the first 3 days of life. On the first assessment of gastric emptying time 11 neonates were on caffeine treatment while the rest did not receive caffeine.

For the second assessment of gastric emptying time neonates were allocated vice versa. Finally, group A consisted of 22 neonates on caffeine treatment and group B consisted of the same neonates without caffeine treatment.

Each neonate during both assessments was under the same type of respiratory support (nasal CPAP or room air). Caffeine citrate was administered in all neonates with GA < 32 weeks after the third day of life (as per our NICU protocol) as a bolus of 20 mg/kg iv, followed by a maintenance dose of 5 mg/kg/day iv or per os when they were on full enteral feeding. The caffeine plasma levels were not routinely measured in our NICU, based on bibliographic data [15,16].

A small pilot study was used to determine the necessary washout period for caffeine after the discontinuation of administration. The three-day break period was considered appropriate as a washout period for assessing gastric emptying time without the effect of caffeine.

For assessing gastric emptying in neonates when they were on caffeine, the drug therapy had to be administered for at least 48 h and for the assessment without caffeine the drug had to be discontinued at least three days prior. During the delayed initial administration of the caffeine in the 11 neonates of group A and the three -days break in neonates of group B, in case of apnea, nasal continuous positive airway pressure (nCPAP) was used for apnea treatment.

Enteral feeding of these neonates started according to the NICU protocol on the second day of life with trophic feeds for approximately three days. It gradually increased by 20–30 mL/kg/day to a maximum amount of 200 mL/kg/day depending on satisfactory weight gain (>15 g/kg/day), based on “aggressive nutrition” strategy, implemented since 2006 in our NICU [17,18]. Gastric residual volume was checked every 4 h and before the next milk feed administration. During assessments, the neonates were receiving at least 80% of their daily needs in milk.

The assessments were always performed in the morning. In every newborn, if the amount of milk had increased during the interval between the 2 measurements, it was reduced for three feeds prior to the second assessment of the gastric emptying in order to match the amount of milk received during the first assessment [19]. The gastrointestinal complications during the 48 h prior to each ACSA assessment were recorded in detail by the nursing staff.

### 2.4. Ultrasound Examination

Gastric emptying time was measured with the ultrasound method proposed by Newell et al. [20]. The Antral Cross Sectional Area (ACSA) assessment was performed in the morning during a two-hour period at the same 7 time-specific points, every 10 min for the first 30 min and thereafter every 30 min; a total of seven measurements were obtained, the last one at 120 min. All care was taken not to disturb the neonates and the probe only touched their abdomen for a few seconds each time. All 44 measurements were performed by the same radiologist, who did not know whether the neonate was on the caffeine treatment or not, using a U/S model Esaote My Lab 50 machine, (Esaote, Genova, Italy) and a 7.5 MHz probe.

### 2.5. Outcome Measures

Gastric emptying time was defined as the half gastric emptying time, the time for ACSA measurement (in cm^2^) to decrease to 50% of its original value, as described by Newell et al., who found that “measurements of ACSA are reproducible, correlate well with gastric volume and fall during gastric emptying in a manner consistent with recognized patterns of gastric emptying” [20].

Regurgitation, emesis, increased gastric residues, bile or blood stained aspirate, visible bowel loops, abdominal distension and hem-positive stools were considered as possible gastrointestinal complications.

### 2.6. Statistical Analysis

The normality of the distribution of the variables was evaluated using statistical tests and diagnostic graphs (Shapiro–Wilks and Kolmogorov–Smirnov tests of normality and P–-P plots and Q–Q plots, correspondingly). Continuous variables are presented as mean and standard deviation if they are normally distributed or median and first and third quartile if their distribution is skewed. Categorical variables are presented as absolute and relative frequencies. Paired Student’s *t*-test was used in order to assess differences in ACSA values between intervention and no-intervention groups. All reported probability values (*p*-values) are based on two sided tests and compared to a significant level of 5%. SPSS 18.0 software was used for all the calculations (SPSS corp., Chicago, IL, USA).

## 3. Results

The clinical profiles and demographic data of the study population are presented in Table 1.

For each group, the first u/s assessment was performed at the median (range) age of 17 (10–31) days of life, for which a mandatory requirement was for the neonates to reach at least 80% of their daily needs in milk, while the second one was performed at 20 (14–34) days of life.

Statistically significant difference was found with regards to the gastric emptying time [median, (range)], as documented by the ultrasound measurement and changes in ACSA values, between caffeine and control group (*p* = 0.040). Furthermore, study neonates were sub classified according to BW (neonates with BW < 1000 g and BW 1000–1500 g) and GA (GA < 28 weeks and GA 28–32 weeks). The gastric emptying time in the neonates with BW 1000–1500 g and GA 28–32 weeks was significantly longer during caffeine treatment than the observed gastric emptying time during no caffeine treatment (*p* = 0.002 and *p* = 0.001, respectively). There was no significant difference in the gastric emptying time of the neonates with BW < 1000 g and GA < 28 weeks (*p* = 0.879 and *p* = 0.964, respectively) (Table 2).

**Figure 2:**
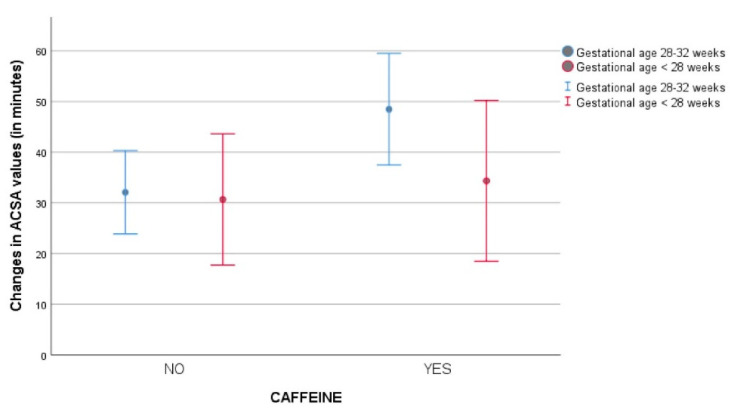
Distribution of Antral Cross Sectional Area (ACSA) values [mean—95% confidence intervals) in the study groups according to gestational age (<28 weeks vs. 28–32 weeks).

Distribution of ACSA values (mean—95% confidence intervals) in the study groups according to gestational age category are presented in Figure 2.

**Figure 3:**
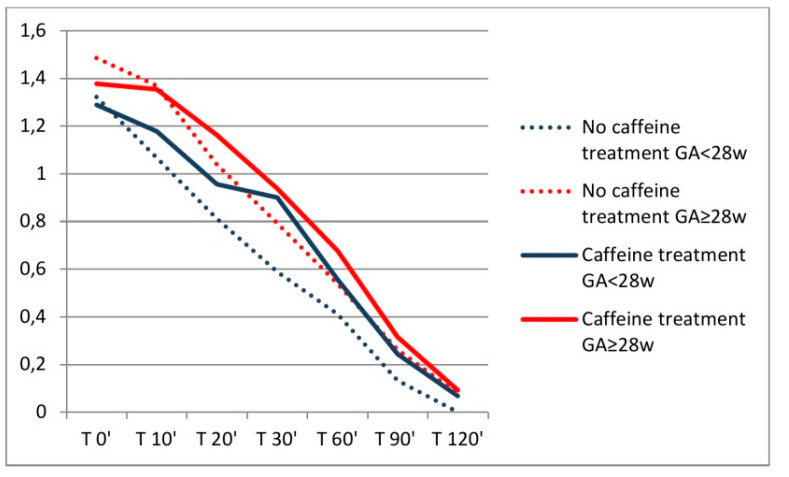
Distribution of ACSA values (means) reduction through different study time points with regards to caffeine treatment and depending on gestational age (GA) in weeks (w).

In Figure 3, ACSA values measured at each time point are presented with regards to gestational age and caffeine intake status. It is noteworthy that at the time point of 120 min, ACSA value was measured in 9 neonates out of 22 being on caffeine treatment and only in 4 neonates out of 22 who did not receive caffeine treatment, while in the remaining neonates in each group ACSA values at 120 min could not be measured as no gastric content was detected.

The incidence of GI complications was significantly greater in neonates receiving caffeine than those without caffeine treatment (*p* = 0.039), while most of these complications were noted in neonates with BW 1000–1500 g during caffeine treatment (*p* = 0.028) (Table 3).

During caffeine administration 4 neonates had increased gastric residue, 1 had regurgitations and 1 abdominal distention. Only 1 neonate presented regurgitation and increased gastric residue in the no caffeine group. In all of the cases an adjustment in milk volume was sufficient and there was no need to stop enteral feeds. No neonate developed NEC during the entire process of the study.

## 4. Discussion

In this study, we documented that caffeine significantly induces delayed gastric emptying in very preterm neonates. According to our study, the caffeine effect depends on neonatal maturity as it increased the gastric emptying time in neonates with GA ≥ 28 weeks and those with BW 1000–1500 g.

Due to the protective effect found in several noteworthy and recently published trials, there is an increase in caffeine use for the treatment of apnea of prematurity in neonatal units [2,5]. This wider use of caffeine raised questions about the possible complications that methylxanthines can generally cause.

The large multicenter CAP trial found no significant difference in the incidence of NEC between the neonates who receive either caffeine or placebo [2].

In a large retrospective study, it was found that early administration of caffeine (before the third day of life) in newborns with BW less than 1250 g can be associated with an increased risk of NEC, raising the possibility of intestinal complications of the drug [21]. Recently, Cox C et al. [22] showed that in infants with BW < 1500 g and GA < 32 w both the use of caffeine and vasopressor significantly increase the risk of developing NEC (*p* = 0.047 and *p* = 0.045, respectively).

Schmidt B et al. [2] found significant reduced weight gain during the first month of life in neonates who received caffeine. Authors concluded that the above finding could be the result of increased oxygen consumption caused by caffeine. Could it also be the result of the caffeine effect on gut motility in neonates?

Bozzetti et al. [23], found that caffeine increases time to reach full enteral feeding in VLBW neonates by seven days (mean, *p* < 0.001). Xu et al. [11], showed that up to 10% of the VLBW neonates have feeding intolerance during caffeine treatment. Likewise, Huang X et al. [24] in a recent study considered that, in the logistic multivariate regression analysis, the use of caffeine was a risk factor for feeding intolerance (*p* < 0.05).

Hence, there is evidence, showing negative effect of caffeine on the gut and especially on gastric motility.

The caffeine treatment dose used in our NICU is considered safe. In two recent papers, high doses of caffeine correlated with a trend for increased incidence of seizures [25] and a decrease in weight gain in the first 60 days of life [26].

An important finding in our study was that caffeine increased gastric emptying time of VP neonates approximately in the third week of life in a statistically significant way. This results from the statistically significant difference in the gastric emptying time that was observed on the subgroup of neonates with BW 1000–1500 g, and those with GA ≥ 28 weeks. As far as complications were concerned, significantly more complications were observed in the caffeine group.

The negative action of the drug that was observed only in our newborns with BW > 1000 g and GA > 28 weeks may be attributed to the different anatomic and functional gastrointestinal properties of this subgroup of neonates. In the literature, drugs may affect gastrointestinal system in a different way according to the stage of maturation. In a study, authors suggested that erythromycin increased gut motility only in infants whose GA was 32 weeks or older, while no effect was documented for earlier GA [27]. Similar results were found by Aly et al. [28] in a randomized study for erythromycin, which demonstrated that the drug had no effect on infants with GA < 32 weeks, but enhanced (increased) gut motility only in infants with GA more than 32 weeks. It appears that certain drugs may have an effect (either positive or negative) only in the GI of more mature infants. Gastric emptying in preterm neonates is affected by several factors such as the type of milk (breast milk or formula), use of a breast milk fortifier, infusion rate of the enteral feeding and the feeding mode (intermittent bolus or continuous) [29]. Ferreira CHF et al. [30] in a study with rats found that the gastric emptying rate is not developmentally regulated, but dependent on the content volume. Contrarily, Ramirez et al. [31], reported that gastric emptying time is related to gestational age at birth even at age one month and not to milk osmolality, volume or density in a study of preterm neonates with GA 25–30 weeks. At the same conclusion came Perella et al. [32] when she studied preterm neonates at 33.3 ± 1.4 weeks postmenstrual age with full enteral feeding.

Our findings that caffeine affected gut motility only on relatively more mature infants may be a reflection of the different degree of maturity of the autonomic neuromuscular system of these infants. Furthermore, as reported by Natarajan et al. [16], preterm neonates with GA 29–32 weeks reach higher caffeine plasma levels compared to those with GA < 29, a fact which may explain our findings. The hypothesis that the caffeine effect may be more obvious in more mature infants due to larger volumes of milk feeds, must be addressed in future studies.

The small number of neonates included in the study is the most important limitation of this study. However, on the other hand, the fact that each newborn underwent measurement of gastric emptying time both on and off caffeine treatment, effectively acting as control of itself and the prolonged U/S observation period of each infant for gastric volume milk changes are felt to be among the strengths of this study. To the best of our knowledge there is no similar study measuring gastric emptying time in VP infants in the international literature.

In conclusion, our study showed that during caffeine treatment a significantly delayed gastric emptying time was observed in neonates with BW 1000–1500 g and those with GA 28–32 weeks. Further and larger studies need to be conducted to confirm these interesting findings.

## Figures and Tables

**Table 1:** Basic characteristics of the study neonates.

Variables	
Number of neonates	22
Birth weight (g) (mean ± SD)	1077 (229)
Gestational age (weeks) (mean ± SD)	28.6 (2.1)
Male (*n*,%)	11 (50)
Antenatal corticosteroids (*n*,%)	12 (54.5)
AGA (*n*,%)	22 (100)
Apgar score (median, range)	
1st min	6 (2–9)
5th min	8 (7–10)
Age of measurements (days)	
(median, range)	
1st	17 (10–31)
2nd	20 (14–34)
Nasal CPAP during measurements (*n*,%)	7 (31.8)
Milk volume during measurements (mL/2 h)	
(mean ± SD)	19.3 (4.8)
(median, range)	18 (12–26)
Mother’s milk with fortifier (*n*,%)	9 (40.9)

Abbreviations: AGA= appropriate for gestational age; CPAP = continuous positive airway pressure.

**Table 2:** Distribution of the half gastric emptying time (MIN) in the study neonates according to their caffeine intake status and the corresponding *p*-value.

	BW Mean (Range)	GA Mean (Range)	No Caffeine *	Caffeine *	*p*-Value
**Total (*n* = 22)**			26 (24–30)	36.5 (25.2–64.2)	0.040
**BW < 1000 g** (*n* = 10) **	864 (770–970)g	26.7 (26–29)w	24.5 (20.7–33)	24.5 (20–47)	0.879
**BW = 1000–1500 g** **(*n* = 12)**	1212 (1020–1440)g	29.3 (28–31)w	27 (24.2–30)	44.5 (36–68.2)	0.002
**GA < 28 w** **(*n* = 9)**	865 (770–970)g	26.4 (26–27)w	24 (20.5–38)	23 (20–54)	0.964
**GA = 28–32 w** (*n* = 13) **	1185 (860–1440)g	29.3 (28–31)w	27 (24.5–30)	40 (34.5–66.5)	0.001

Abbreviations: GA = gestational age; BW = birth weight; w = weeks. * data presented as medians and interquartile ranges in parentheses. ** one neonate was included with BW 860 g and GA 29 weeks.

**Table 3:** Distribution of the presence of any GI complications in the study neonates according to their caffeine intake status and the corresponding *p*-value.

	GI Complication		
	No Caffeine	Caffeine	*p*-Value
**Total (*n* = 22)**	1 (4.6%)	6 (27.7%)	0.039
**BW = 1000–1500 g (*n* = 12)**	1 (8.3%)	4 (33.3%)	0.028
**BW < 1000 g (*n* = 10)**	0	2 (20%)	0.531

Abbreviations: GI = gastrointestinal; BW = birth weight.
